# FT538, iPSC‐derived NK cells, enhance AML cell killing when combined with chemotherapy

**DOI:** 10.1111/jcmm.70169

**Published:** 2025-01-11

**Authors:** Amanda Eckstrom, Anudishi Tyagi, Sajid Mahmood, Lilly Wong, Bahram Valamehr, Adishwar Rao, Akriti Agrawal, Maryam Siddiqui, V. Lokesh Battula

**Affiliations:** ^1^ Department of Leukemia The University of Texas MD Anderson Cancer Center Houston Texas USA; ^2^ Fate Therapeutics, Inc. San Diego California USA; ^3^ Department of Breast Medical Oncology The University of Texas MD Anderson Cancer Center Houston Texas USA

**Keywords:** AML, immunotherapy, iPSC‐derived NK cells, NK cell therapy, NK cell‐mediated apoptosis

## Abstract

Induced pluripotent stem cell (iPSC)–derived natural killer (NK) cells offer an opportunity for a standardized, off‐the‐shelf treatment with the potential to treat a wider population of acute myeloid leukaemia (AML) patients than the current standard of care. FT538 iPSC‐NKs express a high‐affinity, noncleavable CD16 to maximize antibody dependent cellular cytotoxicity, a CD38 knockout to improve metabolic fitness, and an IL‐15/IL‐15 receptor fusion preventing the need for cytokine administration, the main source of adverse effects in NK cell–based therapies. Here, we sought to evaluate the potential of FT538 iPSC‐NKs as a therapy for AML through their effect on AML cell lines and primary AML cells. We observed that FT538 iPSC‐NKs induce effector‐to‐target cell ratio dependent apoptosis in cell lines and primary AML cells, including cells from high‐risk patients. Flow cytometric analysis revealed that FT538 iPSC‐NKs induce AML cell death when combined with the AML therapies: cytarabine, venetoclax and gilteritinib. Moreover, cytarabine did not affect FT538 iPSC‐NK viability, suggesting that iPSC‐derived NK therapies and chemotherapy may be a promising treatment combination. This study provides the basis for further study of iPSC‐derived NK cell therapies as a treatment option for high‐risk AML patients, particularly those with disease resistant to standard therapies.

## 1 INTRODUCTION

Acute myeloid leukaemia (AML) is a fast‐growing, acute cancer characterized by the clonal expansion of blasts in the bone marrow and peripheral blood. It affects myeloid cells—red blood cells, platelets and nonlymphocytic white blood cells (WBCs)—and can result in a weakened immune system, excessive bleeding, ineffective erythropoiesis and bone marrow failure.^1^ AML is the second most common leukaemia in adults, with an incidence rate of 4.1 in every 100,000 people per year.^2^, ^3^

Current treatment options include chemotherapy, radiation therapy, targeted therapy, and bone marrow transplantation.^4^ The 3+7 regimen, which has been the standard of care for AML since the 1970s, is an intensive chemotherapy involving 3 days of daunorubicin followed by 7 days of cytarabine.^5^ However, 10%–40% of patients with newly diagnosed AML never experience complete remission on the 3+7 regimen,^6^ and up to 50% of patients who do achieve complete remission with the 3+7 regimen later relapse.^7^ Bone marrow transplantation is seen as the best chance for a cure after remission,^8^ but even 50% of patients who undergo a bone marrow transplant eventually experience relapse.^9^ Even with recent advances in the treatment of AML with targeted therapies such as the BCL2 inhibitor venetoclax and the CD33 inhibitor gemtuzumab ozogamicin, the prognosis for AML patients remains poor, with a cure rate of only 40% for patients under the age of 60 and as low as 15% for patients 60 years and older,^10^, ^11^ a grim outlook for a disease with a median age of diagnosis of 68 years.^4^

Cell therapies are a type of immunotherapy in which cellular material is injected into the patient. They are most often used in cancer treatment, treatment of immune disorders and regenerative medicine.^12^ Cell therapies have been widely successful in treating hematologic malignancies.^13^ The currently leading approved cell therapies are autologous chimeric antigen receptor (CAR)‐T cell therapies,^14^ but these are expensive, and as many as 30% of patients do not receive the treatment due to the time required to manufacture the cells.^15^ Additional barriers to CAR‐T cell therapies include antigen escape and high rates of treatment‐related toxicity, prompting investigation into alternative cell therapies.^16^

Natural killer (NK) cell–based therapies are increasingly studied due to their advantages of lower treatment‐related toxicity and lack of HLA restriction.^17^ NK cells recognize target cells through the presence of stress markers often found on malignant and virally infected cells, making them an ideal candidate for cancer‐targeting therapies.^18^ Autologous and allogenic NK cell therapies have shown some success in treating resistant AML, with overall response rates of 50%–60% and complete response rates from 40% to 50%.^12^ However, significant challenges, including time and expense, remain before these therapies can be implemented on a clinical scale.^19^ Induced pluripotent stem cell (iPSC)–derived NK cells offer an opportunity for a standardized, off‐the‐shelf treatment that may be cheaper and faster than other cell therapies and treat a wider patient population, but their effect on AML has been unexplored.^17^

FT538 iPSC‐NKs are triple gene edited to express a high‐affinity, noncleavable CD16 that allows them to maintain constant antibody‐dependent cellular cytotoxicity, a CD38 knockout that improves metabolic fitness and prevents fratricide in the presence of a CD38 monoclonal antibody, and an IL‐15/IL‐15 receptor fusion to avoid the need for cytokine administration, a major source of adverse effects from NK cell–based therapies.^20^ In this study, we analysed the effects of FT538 iPSC‐NKs in targeting high‐risk AML that is resistant to standard chemotherapy. Additionally, we determined the anti‐AML effect of FT538 iPSC‐NKs in combination with the approved AML drugs venetoclax, gilteritinib and cytarabine.

## 2 MATERIALS AND METHODS

### 2.1 Cell culture

Kasumi‐1, THP1, MV4‐11 and U937 AML cells were purchased from ATCC (Manassas, VA), and Molm‐13, Molm‐14, OCI‐AML3 and OCI‐AML2 cells were obtained from DSMZ (Braunschweig, Germany). These cell lines were cultured in RPMI1640 media (Corning, 15‐040‐CV) supplemented with 10% foetal bovine serum (FBS) (Gibco, 26140‐079), 1% penicillin/streptomycin (Sigma‐Aldrich, P4333) and 1% L‐glutamine (Corning, 25‐005‐CI). The medium was changed twice weekly. Tests for *Mycoplasma* contamination of leukaemic cells are performed in our laboratory every 4–6 months.

### 2.2 Patient sample characteristics

Primary AML cells were derived from peripheral blood samples collected from 11 patients between February 2023 and April 2023. The sample collection was conducted according to a protocol approved by the Institutional Review Board at The University of Texas MD Anderson Cancer Center (Protocol PA18‐0129), and all study participants provided written informed consent. Patient characteristics were extracted from MD Anderson Cancer Center's electronic health record system (EPIC, Epic Systems Corporation). All participants presented unique cytogenetics and had previously received treatment (Table 1). WBC counts and blast percentages for each patient were recorded by the MD Anderson Leukaemia Sample Bank.

**TABLE 1:** Patient Characteristics (*N* = 11).

Sample	Blast %	WBC (×10^9^ cells/L)	Status at time of collection	AML marker expression	Cytogenetics	Previous treatments*
FTE118	56%	2	Resistant AML	CD7 partial, CD13 partial, CD25 dim, CD34, CD38 decreased, CD45 dim, CD54 partial, CD56, CD117, CD123 partial, CD133 and HLA‐DR decreased	*ASXL1*; *JAK3*; *NF1*; *RUNX1*; *SRSF2*; *TET2*; *TP53* deletion; 17Q insertion; trisomy 13	Cladribine, low‐dose cytarabine, venetoclax, decitabine
FTE294	9%	6.3	Relapsed AML	CD123, CD4, CD7 partial, CD13 positive, CD25 partial, CD33 increased	*GATA2*; *KRAS*	High‐dose cytarabine, hydroxyurea, cladribine, idarubicin, venetoclax
FTE644	7%	9	Primary refractory AML	CD4 partial, CD5 partial, CD13, CD15, CD19, CD22, CD25 partial, CD33, CD38 decreased, CD45 dim, CD64, CD71, CD117 increased, CD123 increased, HLA‐DR partial, MPO partial, TDT	*ASXL1*; *DNMT3A*; *FLT3*; *PHF6*; *RUNX1*; *NRAS*; trisomy 13	Venetoclax, cytarabine, cladribine, CPX‐GO, stem cell transplant
FTE855	78%	20.6	Resistant AML	CD13, CD33, CD38 decreased, CD45 dim, CD71 partial, CD117 bright, CD123, HLA‐DR, MPO	*DNMT2A*; *IDH2*; *NPM1*; *WT1*	Venetoclax, cladribine, low‐dose cytarabine, azacitidine, decitabine, cedazuridine and enasidenib, hydroxyurea
FTE193	72%	11.4	AML not having reached complete remission	CD34^+^, CD38^+^, CD45 dim, CD64 partial, CD117, HLA‐DR	*DNMT3A*; *FLT3*; *IDH1*; *NRAS*; *SF3A1*	HM43239, venetoclax, gilteritinib, cladribine, decitabine
FTE575	86%	27.3	Resistant AML	CD4 dim, CD13, CD33, CD34, CD36 partial, CD38 decreased, CD45 dim, CD54, CD117, CD123, CD133, HLA‐DR	*TP53*; *U2AF1*	Hydroxyurea, cytarabine, decitabine, cladribine, venetoclax
FTE178	12%	1.8	Resistant AML	CD4 increased, CD13 partial, CD33, CD34 partial, CD36 bright, CD38 decreased, CD45, CD54 increased, CD117, CD123 partial, HLA‐DR partial	*CUX1*; *TP53*	CPX‐GO, cladribine, cytarabine, azacitidine, venetoclax, daunorubicin
FTE888	93%	4.4	Relapsed AML	CD4 partial, CD13, CD15 partial, CD33, CD34, CD36, CD38 increased, CD45 decreased, CD54 partial, CD64 partial, CD117 increased, CD123 increased, CD133 decreased, HLA‐DR decreased, MPO, TDT partial	*FLT3*; *WT1*; *TET2*; *NRAS*; *PTPN11*; trisomy 8	GO, decitabine, midostaurin, venetoclax, gilteritinib, cyclophosphamide, mesna, VP16, carboplatin, cytarabine, stem cell transplant
FTE999	34%	14.3	AML not having reached complete remission	CD13 increased, CD33 partial, CD34, CD36 partial, CD38 decreased, CD45 dim, CD54, CD117 increased, CD123 increased, CD133, HLA‐DR, MPO	*FLT3*; *MLP*; *SRSF2*; *TET2*	Cladribine, low‐dose cytarabine, venetoclax, azacitidine
FTE514	87%	21.8	AML not having reached complete remission	CD13, CD33, CD34, CD38, CD45 dim, CD117 partial, CD123, HLA‐DR, MPO	*CUX11*; *JAK2*; *NF1*; *SMC1A*; *SRSF2*; *TET2*; *TP53*	Azacitidine, cladribine, idarubicin, cytarabine, venetoclax, hydroxyurea
FTE045	54%	27.2	AML not having reached complete remission	CD4 partial, CD13, CD14, CD15 partial, CD33, CD36, CD38, CD45, CD54, CD56 increased, CD64, CD123 partial, HLA‐DR	*ASXL1*; *KRAS*; *TET2*	Cladribine, low‐dose cytarabine, venetoclax

Abbreviations: CPX‐GO, CPX‐351 and gemtuzumab ozogamicin; WBC, white blood cell count.

* Previous treatments received outside of MD Anderson Cancer Center may not be available for all patients.

### 2.3 Primary AML cell isolation

AML peripheral blood mononuclear cells (AML‐PBMCs) were isolated from patient peripheral blood samples using density grade centrifugation. The viability and number of the PBMCs were measured with a Beckman Coulter Vi‐CELL XR cell counter, and only samples with a viability above 85% were selected for live cell imaging apoptosis assays. The AML‐PBMCs from six samples were used immediately for apoptosis assays, and AML‐PBMCs from five samples were frozen before use. Frozen samples were stored in liquid nitrogen in a solution of 10% dimethyl sulfoxide and 90% foetal bovine serum (Gibco, 26140‐079) that was washed off immediately upon thawing.

### 2.4 Generation of FT538 iPSC‐NKs

We used our nonintegrating plasmid system to reprogram donor fibroblasts into iPSC lines.^21^ The FT538 iPSC line was generated as previously described by Woan et al.^20^ In short, the FT538 iPSCs were genetically modified using the CRISPR/Cas9 system by targeting IL‐15RF and hnCD16 to the CD38 locus. Clonal iPSC lines underwent screening to ensure precise knock‐in and knockout edits at the CD38 locus, confirm the absence of reprogramming transgenes, and verify the stability of the genome. Furthermore, these engineered FT538 iPSC clones underwent testing for their NK cell differentiation potential and functionality.

### 2.5 Co‐Culture and live cell imaging apoptosis assays

FT538 iPSC‐NKs were co‐cultured with AML cells in B0 medium made with 60% Dulbecco's modified Eagle's medium (Corning, 10‐017‐CV) with 30% Ham's F‐12 medium (Corning, 10‐080‐CV), 10% Human‐AB Serum (IVT, HP1022I), 1% penicillin/streptomycin (Sigma‐Aldrich, P4333), 1% HEPES solution (Corning, 25‐060‐CI) and 1% 100X B0 supplement mix (Gibco, ME18527L1). AML cells from 8 AML cell lines and primary AML cells derived from 11 patients were stained with cytolyte red (Essen BioScience, 4706) prepared according to the manufacturer's recommendations.^22^ The stained AML cells were seeded on a flat‐bottom 96‐well plate (Falcon, 353072) at a density of 5 × 10^4^ cells in 50 μL of B0 medium per well (1 × 10^6^ cells/mL) and incubated for 30 min. Then media for the untreated control and FT538 iPSC‐NKs at effector‐to‐target cell (E:T) ratios of 2:1, 4:1 and 8:1 were added to the plate. The whole plate was stained with Annexin V green (Sartorius, 4642) prepared according to the manufacturer's instructions. The co‐culture was scanned for live AML cells every hour for 24 h using the Sartorius IncuCyte Live Cell Analysis System, with four images taken of each well for each time point. Apoptosis in AML cells was detected by the overlap of the green (apoptotic cells) and red (AML cells) fluorescence signals.

### 2.6 Flow cytometric analysis of combination treatments

To assess the apoptosis induction by FT538 iPSC‐NKs in combination with approved AML therapies, AML cell lines were treated with various doses of venetoclax, gilteritinib, cytarabine or a B0 media vector for 48 h in 96‐well flat‐bottom plates (Falcon, 353072) before being cultured alone or co‐cultured with FT538 iPSC‐NKs at a 2:1 E:T ratio overnight for 18 h. Each sample was then transferred to a round‐bottom plate (Corning, 3799) for staining with CD33‐PE (Biolegend, 303404), NKG2D‐APC (Biolegend, 320808), Annexin V‐FITC (Biolegend, 640945) and 0.25 μg/mL DAPI (Invitrogen, 1890543) in Annexin V binding buffer (Biolegend, 422201). Flow cytometric analysis was performed using a Beckman Coulter CytoFlex flow cytometer.

### 2.7 Statistical analysis

The data from live cell imaging apoptosis assays and flow cytometry assays were reported as mean ± standard error. AML cell apoptosis rates were compared between treatment groups by one‐way Welch's ANOVA with multiple comparisons. Peak apoptosis rates for each E:T ratio across all AML cell lines were compared using two‐way ANOVA with multiple comparisons. Spearman's rank order correlation was used to assess correlations between the blast percentages and WBC counts of the patient samples with their sensitivity to FT538 iPSC‐NKs, as both the peak apoptosis signal measured and the time of the peak. All figures and analyses were generated using GraphPad Prism 10, except for flow cytometry dot plots, which were generated using FlowJo v10.10.0 software. *p* values lower than 0.05 were considered significant.

## 3 RESULTS

### 3.1 FT538 iPSC‐NKs potently induce apoptosis in AML cell lines

**FIGURE 1:**
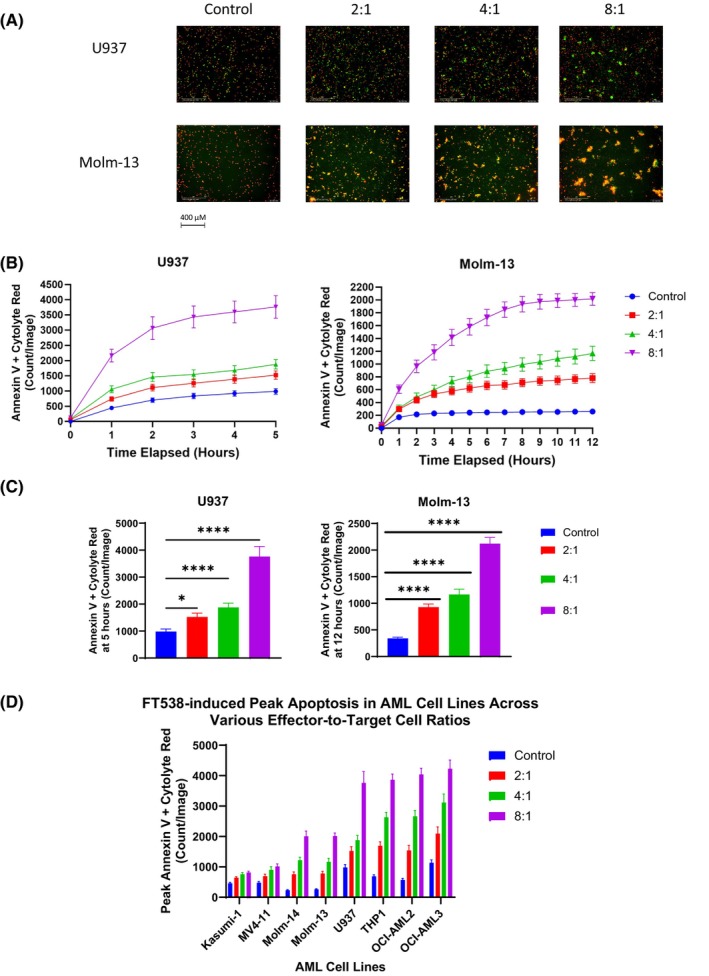
FT538 iPSC‐NKs induce apoptosis in AML cell lines. (A) Representative live cell images showing apoptosis in AML cell lines. AML cells were labelled with cytolyte red, and apoptotic cells with Annexin V green. AML cell apoptosis was quantified as the overlap between the red and green fluorescence signals. (B) Time series graphs of apoptosis in U937 and Molm‐13 cell lines after co‐culture with FT538 iPSC‐NK cells. (C) Bar graphs comparing peak apoptosis levels for each effector‐to‐target cell ratio in U937 and Molm‐13 AML cell lines. Welch's one‐way anova with multiple comparisons was used to determine significance. (D) Comparison of peak apoptosis levels for 2:1, 4:1 and 8:1 effector‐to‐target cell ratios across 8 AML cell lines. *p < 0.05, **p < 0.01, ***p < 0.001, ****p < 0.0001.

To investigate the effect of FT538 iPSC‐NKs on AML cell lines, we performed live cell imaging apoptosis assays on AML cell lines co‐cultured with FT538 iPSC‐NKs. Representative images of AML cell lines undergoing FT538 iPSC‐NK–induced apoptosis at the peak time points for each E:T ratio are shown in Figure 1A, Figure S1A. Time series analysis indicated that apoptosis induction by FT538 iPSC‐NKs in all of the tested AML cell lines was dependent on time and E:T ratio (Figure 1B, Figure S1B). At their peak (5 h), U937 cells exhibited the highest rate of apoptosis at the 8:1 E:T ratio (*p* < 0.0001 vs. untreated cells), and apoptosis rates were also significantly higher than in the untreated cells at E:T ratios of 4:1 (*p* < 0.0001) and 2:1 (*p* < 0.05) (Figure 1C). The Molm‐13 cell line, which carries an *FLT3* mutation, reached peak apoptosis at the 12‐h time point (*p* < 0.0001) (Figure 1C).

Apoptosis rates at the peak time points for all cell lines are shown in Figure S1C. Comparison of the cell lines' peak sensitivity to FT538 iPSC‐NK–induced apoptosis showed that OCI‐AML3 was the most sensitive cell line. In addition, the peak apoptosis level for each E:T ratio was significantly different from controls in all cell lines (*p* < 0.0001; Figure 1D). However, FT538 iPSC‐NK–mediated apoptosis rates significantly differed between AML cell lines (*p* < 0.0001), indicating that the cell lines had different levels of sensitivity to FT538 iPSC‐NKs. In all cell lines, the rate of apoptosis at the peak was associated with E:T ratio (*p* < 0.0001) (Figure 1D). Overall, these findings demonstrate that FT538 iPSC‐NKs potently induce apoptosis in AML cell lines representing a variety of AML subtypes and cytogenetic presentations, including those with *FLT3* (Molm‐13, Molm‐14, MV4‐11) and *TP53* mutations (Kasumi‐1).

### 3.2 FT538 iPSC‐NKs induce apoptosis in primary AML cells

**FIGURE 2:**
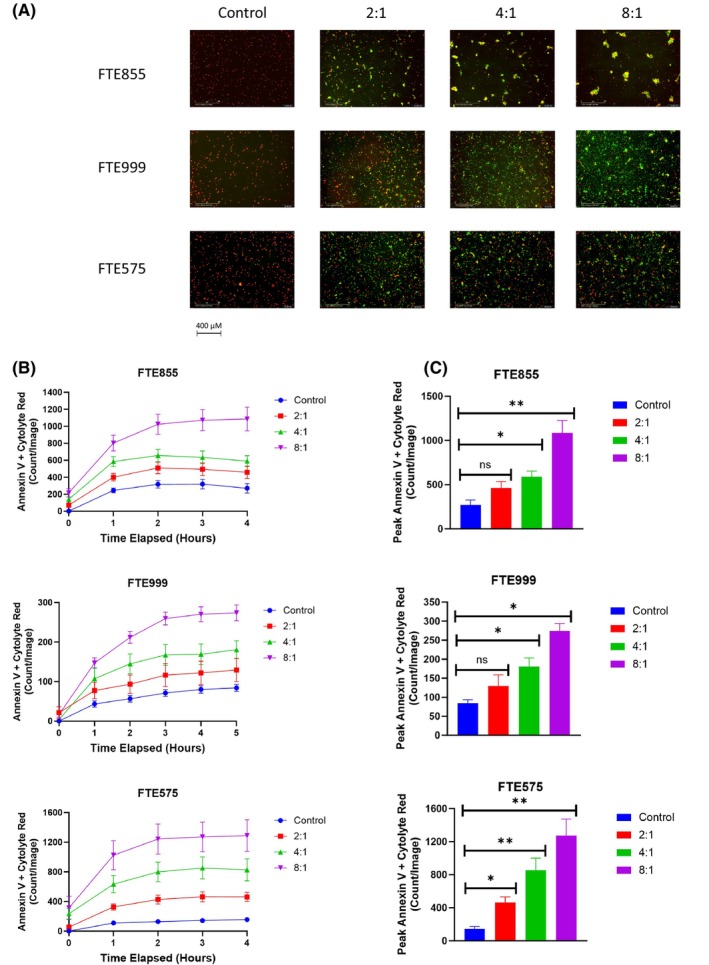
FT538 iPSC‐NKs induce apoptosis in primary AML cells. (A) Representative live cell images showing apoptosis in primary AML cells with wild‐type *FLT3* and *TP53* (FTE855), with *FLT3* mutation (FTE999) and with *TP53* mutation (FTE575). Primary AML cells were labelled with cytolyte red, and apoptotic cells were labelled with Annexin V green. AML cell apoptosis was quantified as the overlap of the red and green fluorescence signals. (B) Time series graphs of FT538 iPSC‐NK–induced apoptosis of primary AML cells in patient samples with or without *FLT3* and *TP53* mutations. (C) Bar graphs comparing peak FT538 iPSC‐NK–induced apoptosis levels for 2:1, 4:1 and 8:1 effector‐to‐target cell ratios for each AML patient sample. Welch's one‐way ANOVA with multiple comparisons was used to determine significance. *p < 0.05, **p < 0.01, ***p < 0.001, ****p < 0.0001, ns, not significant.

Next, we evaluated the effect of FT538 iPSC‐NKs on primary AML cells isolated from 11 different patients. Patient characteristics are shown in Table 1, and mutations present in more than one patient are shown in Table S1. The FT538 iPSC‐NKs robustly induced apoptosis in an E:T ratio‐dependent manner in primary AML cells from all 11 patient samples (Figure S2), including primary AML cells derived from the four patients with *FLT3* mutations and three with *TP53* mutations (Figure 2, Figure S2). Figure 2A shows representative images of FT538 iPSC‐NK–induced apoptosis in primary AML cells at the 4‐hour time point. Further, we found that FT538 iPSC‐NK–mediated apoptosis was dependent on time and E:T ratio in all of the tested primary AML cells (Figure 2C, Figure S2B), including primary AML cells isolated from patients with treatment‐resistant AML. Interestingly, we found significantly greater apoptosis in primary AML cells that were co‐cultured with FT538 iPSC‐NKs at an 8:1 ratio (*p* < 0.01) and a 4:1 ratio (*p* < 0.05) compared to the untreated primary AML cells (Figure 2C, Figure S2B). No correlation was observed between blast percentage or WBC count and peak apoptosis level or peak apoptosis time (data not shown). In contrast to the cell line findings, we found no correlation between the induction of apoptosis by FT538 iPSC‐NKs and the primary AML cells' mutation profiles (including *FLT3* and *TP53* mutations). However, the apoptosis‐inducing effect of FT538 iPSC‐NKs in primary AML cells was dependent on the E:T ratio across the patients' cytogenetic backgrounds, including those with treatment‐resistant disease (Table 1).

### 3.3 FT538 iPSC‐NKs are effective in combination with approved AML therapies

**FIGURE 3:**
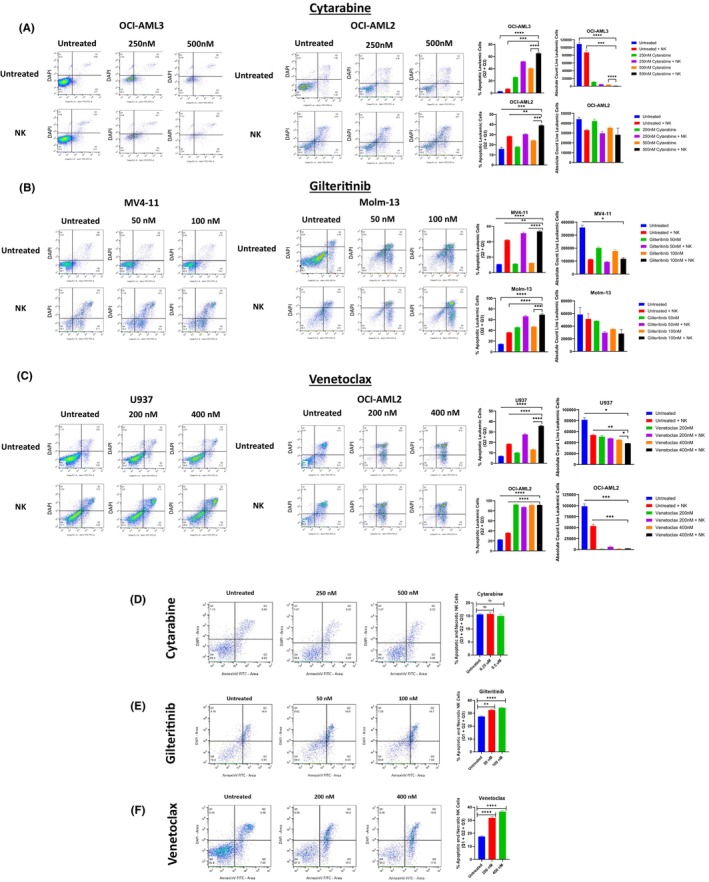
FT538 iPSC‐NKs are effective in combination with approved AML therapies. Flow cytometry was used to determine the combinatorial effects of FT538 iPSC‐NKs with approved AML therapies. NK cells were separated from AML cells via NKG2D‐APC and CD33‐PE staining, Annexin V‐FITC was used as an apoptosis marker, and cell viability was determined with DAPI. (A) Flow cytometry plots of apoptosis in OCI‐AML3 and OCI‐AML2 cell lines treated as indicated with cytarabine + FT538 iPSC‐NKs and bar graphs showing the percentage of apoptotic leukaemic cells and the absolute count of live leukaemic cells. (B) Flow cytometry plots of apoptosis in MV4‐11 and Molm‐13 cell lines treated as indicated with gilteritinib + FT538 iPSC‐NKs and bar graphs showing the percentage of apoptotic leukaemic cells and the absolute count of live leukaemic cells. (C) Flow cytometry plots of apoptosis in U937 and OCI‐AML3 cell lines treated as indicated with venetoclax + FT538 iPSC‐NKs and bar graphs showing the percentage of apoptotic leukaemic cells and the absolute count of live leukaemic cells. (D–F) Flow cytometry plots and bar graphs showing the viability of FT538 iPSC‐NKs after 18 h in the presence of cytarabine, gilteritinib or venetoclax. Significance was determined through Welch's one‐way ANOVA. *p < 0.05, **p < 0.01, ***p < 0.001, ****p < 0.0001.

Because FT538 iPSC‐NK treatment would be more clinically relevant and accessible to patients if used in combination with currently approved AML therapies, we next determined the effect of the combination of FT538 iPSC‐NKs with cytarabine, gilteritinib and venetoclax. The combination of cytarabine and FT538 iPSC‐NKs (cytarabine + FT538) induced dose‐dependent AML cell death, as shown through the absolute live leukaemic cell count (*p* < 0.05) in OCI‐AML3 cells and the percentage of apoptotic cells (*p* < 0.0001) in OCI‐AML3 and OCI‐AML2 cells (Figure 3A). Further, the OCI‐AML3 cells treated with 500 nM cytarabine + FT538 had a significantly lower absolute live leukaemic cell count compared to cells treated with 500 nM cytarabine alone (*p* < 0.0001) or FT538 iPSC‐NKs alone (*p* = 0.0003). Similarly, we observed a significantly higher percentage of apoptotic OCI‐AML3 cells when treated with 500 nM cytarabine + FT538 compared to cytarabine alone (*p* < 0.0001) or FT538 iPSC‐NKs alone (*p* = 0.0001). We further validated these findings in OCI‐AML2 cells, observing higher rates of apoptosis in AML cells treated with cytarabine + FT538 compared to cytarabine alone (*p* = 0.0002) or FT538 iPSC‐NKs alone (*p* = 0.0011) (Figure 3A).

We then sought to assess the combination effect of FT538 iPSC‐NKs with the FLT3 inhibitor gilteritinib. The percentage of apoptotic MV4‐11 cells, which carry an *FLT3* mutation, was significantly higher with the gilteritinib (100 nM) + FT538 combination treatment than with gilteritinib (*p* < 0.0001) or FT538 iPSC‐NKs (*p* < 0.01) alone, and the absolute live cell count was significantly lower among MV4‐11 cells treated with the combination (*p* < 0.05) compared to those treated with gilteritinib or FT538 iPSC‐NKs alone (Figure 3B). Similar findings were observed in Molm‐13 cells, which also carry an *FLT3* mutation (Figure 3B).

Finally, we examined the effect of combining the BCL2 inhibitor venetoclax and FT538 iPSC‐NKs in U937 and OCI‐AML2 cell lines. We found that the percentage of apoptotic U937 cells was significantly higher in the combination‐treated cells than in those treated with 400 nM venetoclax (*p* < 0.0001) or FT538 iPSC‐NKs alone (*p* < 0.0001) (Figure 3C). The absolute live cell count was also significantly lower in U937 cells treated with 400 nM venetoclax + FT538 compared to those treated with either venetoclax alone (*p* < 0.05) or FT538 iPSC‐NKs alone (*p* < 0.01) (Figure 3C). Similar results were observed in the OCI‐AML2 cell line. These results demonstrated that FT538 iPSC‐NK combinations with cytarabine, gilteritinib and venetoclax are highly effective against leukaemic cells, including *FLT3*‐mutated AML cell lines for the gilteritinib combination.

### 3.4 FT538 iPSC‐NK cell viability is not hindered by cytarabine

To evaluate the clinical potential of each combination treatment, it was also important to consider the effects of gilteritinib, venetoclax and cytarabine on the viability of the FT538 iPSC‐NK cells themselves. We discovered that venetoclax (*p* < 0.0001) and gilteritinib (*p* < 0.0001) significantly inhibited the viability of the FT538 iPSC‐NKs (Figure 3E,F). However, we noted that cytarabine had no significant effect on the viability of the FT538 iPSC‐NKs (Figure 3D). These findings suggest that combining FT538 iPSC‐NK therapy with cytarabine may be a particularly promising avenue for AML treatment.

## 4 DISCUSSION

In this study, we investigated the therapeutic potential of FT538 iPSC‐NKs as an off‐the‐shelf treatment platform for high‐risk AML, including treatment‐resistant AML. Our study revealed that FT538 iPSC‐NK cells induced apoptosis in all eight AML cell lines tested. These results align with recent studies reporting that iPSC‐derived NK cell therapies were effective against a variety of cancers including hematologic malignancies.^20^, ^23^ We also observed that the *FLT3* wild‐type OCI‐AML3 cell line was the most sensitive to FT538 iPSC‐NK–mediated killing, whereas the *FLT3*‐mutated MV4‐11 cell line was among the least sensitive. Because all 3 *FLT3*‐mutant cell lines were less sensitive to the FT538 iPSC‐NK therapy, we speculated that the *FLT3* mutation might account for this difference. The anti‐AML effects of FT538 iPSC‐NKs were further supported in primary AML cells. FT538 iPSC‐NKs induced apoptosis in all samples in an E:T ratio–dependent manner. This was particularly noteworthy because our patient cohort included several patients with relapsed/refractory and treatment‐resistant AML. However, no correlation was observed between mutation status and the apoptotic effect of the FT538 iPSC‐NKs in our patient cohort, perhaps because of the small number of samples with each mutation.

A recent study demonstrated that FT538 iPSC‐NKs in combination with daratumumab (an anti‐CD38 monoclonal antibody) are highly effective against multiple myeloma.^20^ Therefore, we sought to determine the effects of FT538 iPSC‐NKs in combination with approved AML therapies. Combining FT538 iPSC‐NK cells with the FLT3 inhibitor gilteritinib resulted in a higher rate of AML cell apoptosis in the *FLT3*‐mutant cell lines Molm‐13 and MV4‐11 than treating with the FT538 iPSC‐NKs alone. This result parallels the finding of Li et al. that combining gilteritinib with bispecific FLT3 single chain variable fragment/NKG2D‐CAR T cells had an enhanced antileukemic effect.^24^ Additionally, we found similar combinatorial effects with venetoclax and cytarabine. The combination of FT538 iPSC‐NK cells with venetoclax could be further enhanced in future studies by incorporating a BCL2 G101V mutation, which was recently reported to induce venetoclax resistance in NK cells.^25^ It is worth noting that the cytarabine + FT538 iPSC‐NK combination was effective in OCI‐AML2 and OCI‐AML3 cells, which are resistant to cytarabine.^26^ Furthermore, cytarabine targets proliferating cells during DNA synthesis,^27^ and it was reported that the activation of CD16a induces NK cell proliferation^28^; therefore, it was interesting that cytarabine did not affect the viability of the FT538 iPSC‐NK cells. However, cytarabine has previously been associated with increased NKG2D ligand expression on AML cells.^29^ This effect has been shown by various chemotherapeutic agents,^30^ as well as in cytarabine‐resistant AML cells.^29^ This indicates that combining FT538 iPSC‐NK therapy with cytarabine and other chemotherapeutic agents may be a promising avenue for AML treatment, particularly in treating cytarabine‐resistant AML.

In conclusion, our study demonstrates the potential of FT538 iPSC‐NK therapy as a treatment option for high‐risk AML. We found that FT538 iPSC‐NKs induced apoptosis in all AML cell lines and primary AML cells and were effective in combination with the approved AML drugs gilteritinib, venetoclax and cytarabine. Additionally, FT538 iPSC‐NK cell viability was unaffected by cytarabine, indicating that combining FT538 iPSC‐NKs with cytarabine may be a promising avenue to treat cytarabine‐resistant AML. Altogether, this study highlights the promising treatment option that FT538 iPSC‐NK therapy may offer for patients with relapsed, refractory, and treatment‐resistant AML.

## AUTHOR CONTRIBUTIONS

**Amanda Eckstrom:** Data curation (lead); formal analysis (lead); investigation (lead); methodology (equal); project administration (equal); resources (lead); validation (equal); visualization (lead); writing – original draft (lead); writing – review and editing (equal). **Anudishi Tyagi:** Data curation (supporting); formal analysis (supporting); investigation (supporting); methodology (equal); project administration (equal); validation (equal); visualization (supporting); writing – review and editing (equal). **Sajid Mahmood:** Conceptualization (equal); methodology (equal); writing – review and editing (equal). **Lilly Wong:** Conceptualization (equal); methodology (equal); writing – review and editing (equal). **Bahram Valamehr:** Conceptualization (equal); methodology (equal); writing – review and editing (equal). **Adishwar Rao:** Writing – original draft (supporting). **Akriti Agrawal:** Writing – original draft (supporting). **Maryam Siddiqui:** Investigation (supporting); resources (supporting). **V. Lokesh Battula:** Conceptualization (equal); funding acquisition (lead); project administration (equal); supervision (lead); writing – review and editing (lead).

## FUNDING INFORMATION

This work was supported by funding from Fate Therapeutics and from the National Institutes of Health/National Cancer Institute through a Cancer Center Support Grant (P30CA016672) to The University of Texas MD Anderson Cancer Center (used the Flow Cytometry and Cellular Imaging Core Facility).

## CONFLICT OF INTEREST STATEMENT

Sajid Mahmood, Lilly Wong, and Bahram Valamehr are employees of Fate Therapeutics, Inc.

## Supporting information


**Figure S1.** FT538 iPSC‐NKs induce apoptosis in AML cell lines. (A) Representative live cell images showing apoptosis in OCI‐AML2, OCI‐AML3, U937, THP1, Molm‐13, Molm‐14, MV4‐11 and Kasumi‐1 AML cell lines. (B) Time series graphs of apoptosis in the indicated cell lines after co‐culture with FT538 iPSC‐NK cells. (C) Bar graphs comparing peak FT538 iPSC‐NK‐induced apoptosis levels at 2:1, 4:1 and 8:1 effector‐to‐target cell ratios in each AML cell line. Welch’s one‐way ANOVA with multiple comparisons was used to determine significance. *p < 0.05, **p < 0.01, ***p < 0.001, ****p < 0.0001.
**Figure S2.** FT538 iPSC‐NKs induce apoptosis in primary AML cells. (A) Time series graphs showing apoptosis in primary AML cells from 11 AML patients after co‐culture with FT538 iPSC‐NK cells. (B) Bar graphs comparing peak FT538 iPSC‐NK–induced apoptosis levels for 2:1, 4:1 and 8:1 effector‐to‐target cell ratios for each patient sample. Welch’s one‐way ANOVA with multiple comparisons was used to determine significance. *p < 0.05, **p < 0.01, ***p < 0.001, ****p < 0.0001, ns, not significant.

**Table S1.** Patient sample characteristics by common mutation status.

## Data Availability

The data that support the findings of this study are available from the corresponding author upon reasonable request.
