# Supplementary material for: FT538, iPSC‐derived NK cells, enhance AML cell killing when combined with chemotherapy
Source: J Cell Mol Med. 2025 Jan 11;29(1):e70169. doi: 10.1111/jcmm.70169 (PMC11724334; doi:10.1111/jcmm.70169)

Supplementary Figure 1.

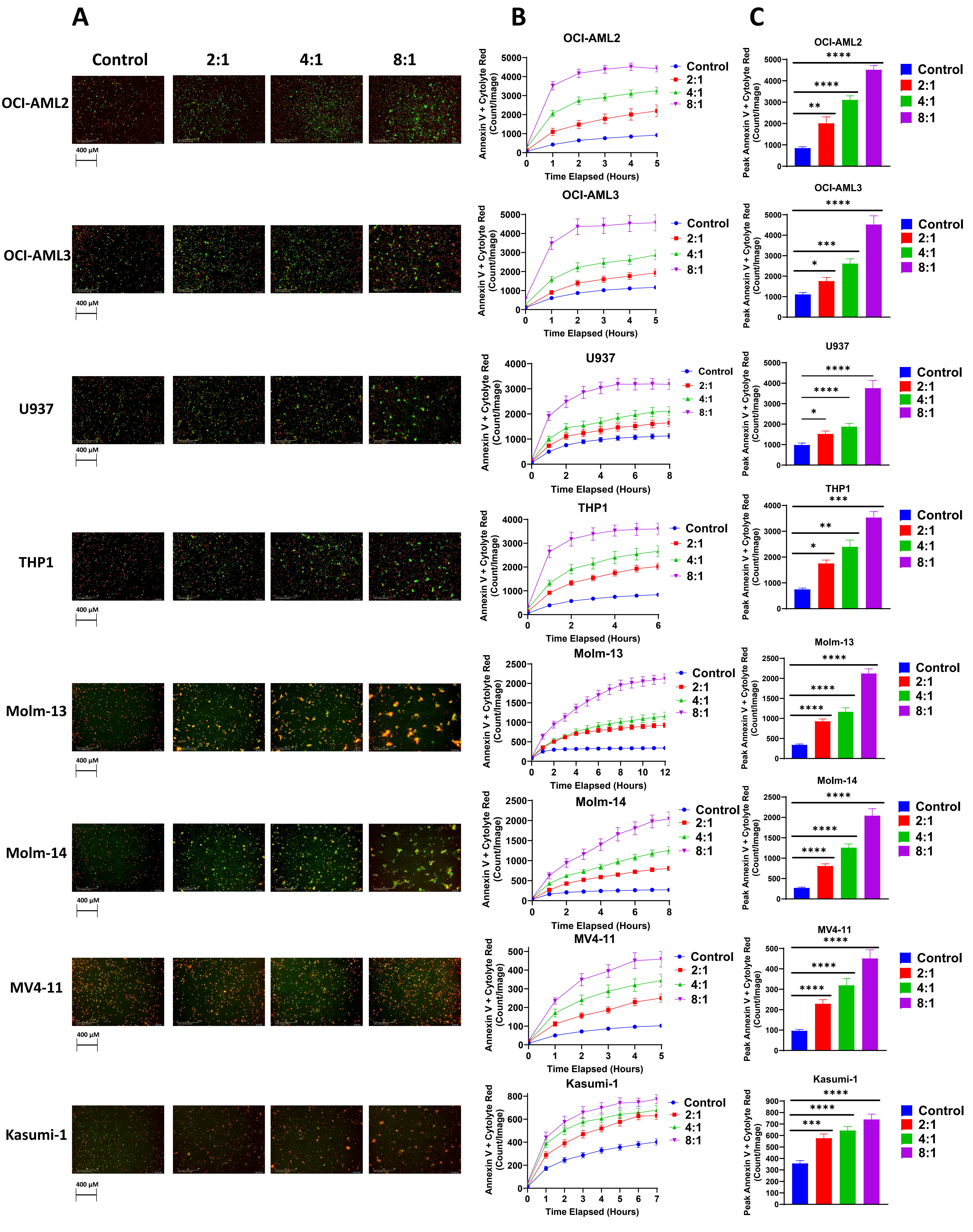

Supplementary Figure 2.

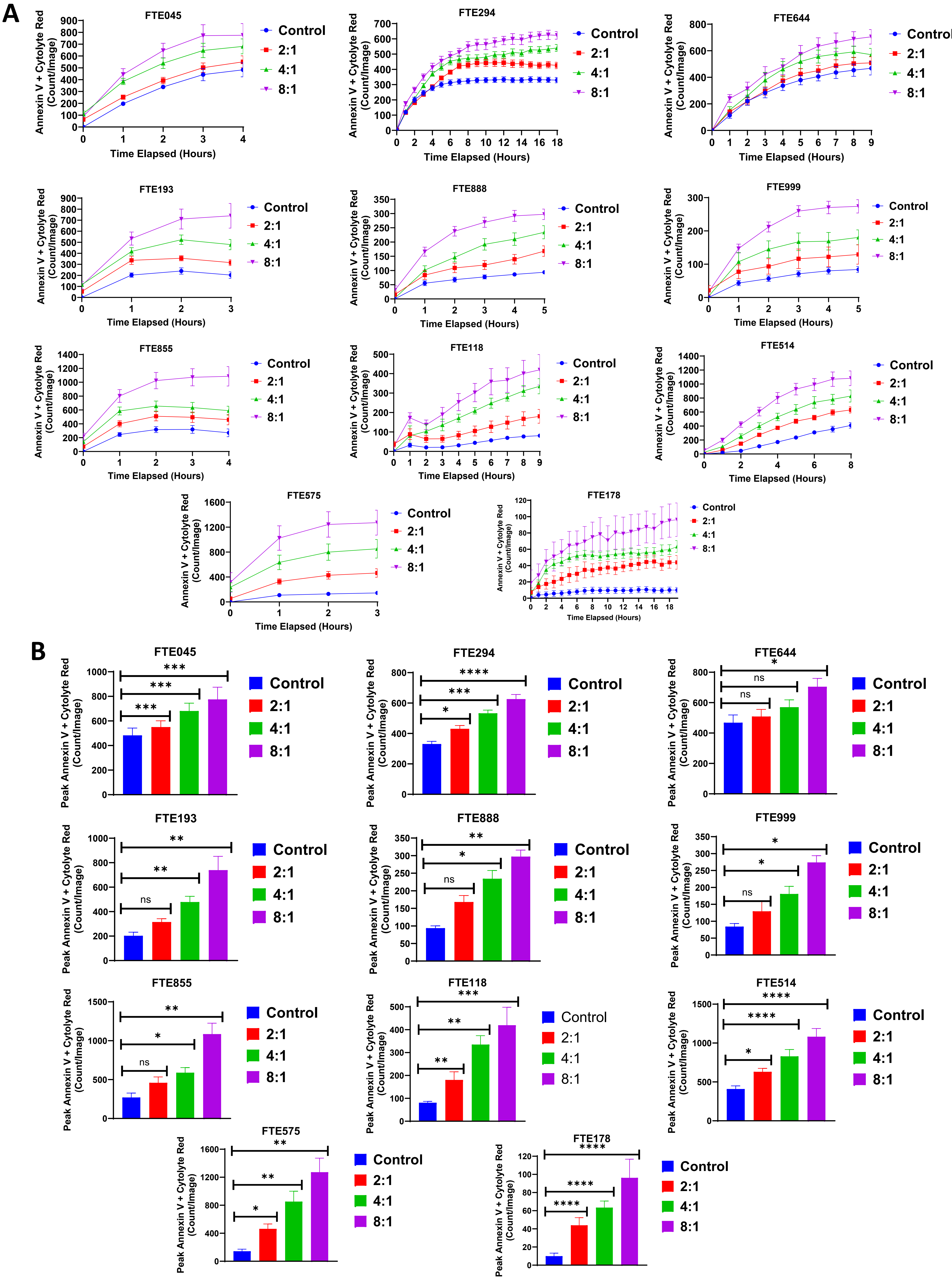

Supplement: Supplementary file 1 — Figure S1. FT538 iPSC‐NKs induce apoptosis in AML cell lines. (A) Representative live cell images showing apoptosis in OCI‐AML2, OCI‐AML3, U937, THP1, Molm‐13, Molm‐14, MV4‐11 and Kasumi‐1 AML cell lines. (B) Time series graphs of apoptosis in the indicated cell lines after co‐culture with FT538 iPSC‐NK cells. (C) Bar graphs comparing peak FT538 iPSC‐NK‐induced apoptosis levels at 2:1, 4:1 and 8:1 effector‐to‐target cell ratios in each AML cell line. Welch’s one‐way ANOVA with multiple comparisons was used to determine significance. *p < 0.05, **p < 0.01, ***p < 0.001, ****p < 0.0001. Figure S2. FT538 iPSC‐NKs induce apoptosis in primary AML cells. (A) Time series graphs showing apoptosis in primary AML cells from 11 AML patients after co‐culture with FT538 iPSC‐NK cells. (B) Bar graphs comparing peak FT538 iPSC‐NK–induced apoptosis levels for 2:1, 4:1 and 8:1 effector‐to‐target cell ratios for each patient sample. Welch’s one‐way ANOVA with multiple comparisons was used to determine significance. *p < 0.05, **p < 0.01, ***p < 0.001, ****p < 0.0001, ns, not significant. [file JCMM-29-e70169-s001.pdf]
