# Supplementary material for: FT538, iPSC‐derived NK cells, enhance AML cell killing when combined with chemotherapy
Source: J Cell Mol Med. 2025 Jan 11;29(1):e70169. doi: 10.1111/jcmm.70169 (PMC11724334; doi:10.1111/jcmm.70169)
Supplement: Supplementary file 2 — Table S1. Patient sample characteristics by common mutation status. [file JCMM-29-e70169-s002.docx]

**Supplementary Table 1. Patient sample characteristics by common mutation status.**

| **Mutation** | **Sample** | **Blast%** | **WBC (K/uL)** | **Additional Mutations** | **Peak Apoptosis (hours)** | **Annexin V (+) cells/field (8:1)** |
| --- | --- | --- | --- | --- | --- | --- |
| *TET2* | FTE118 | *56* | 2 | ASXL1; JAK3; NF1; RUNX1; SRSF2 | 9 | 419.92 |
|  | FTE888 | *93* | 12.1 | FLT3; WT1 | 5 | 297.38 |
|  | FTE999 | *15* | 13.7 | FLT3; MLP; SRSF2 | 5 | 273.90 |
|  | FTE514 | *8* | 14.7 | CUX11; JAK2; NF1; SMC1A; SRSF2; TP53 | 8 | 1081.92 |
|  | FTE045 | *5* | 11.5 | ASXL1; KRAS | 4 | 775.25 |
| *FLT3* | FTE644 | *12* | 8.2 | ASXL1; DNMT3A; PHF6; RUNX1 | 9 | 704.75 |
|  | FTE193 | *75* | 8.9 | DNMT3A; IDH1; NRAS; SF3A1 | 3 | 739.50 |
|  | FTE888 | *93* | 12.1 | WT1; TET2 | 5 | 297.38 |
|  | FTE999 | *15* | 13.7 | MLP; SRSF2; TET2 | 5 | 273.90 |
| *ASXL1* | FTE118 | *56* | 2 | JAK3; NF1; RUNX1; SRSF2; TET2 | 9 | 419.92 |
|  | FTE644 | *12* | 8.2 | DNMT3A; FLT3; PHF6; RUNX1 | 9 | 704.75 |
|  | FTE045 | *5* | 11.5 | KRAS; TET2 | 4 | 775.25 |
| *SRSF2* | FTE118 | *56* | 2 | ASXL1; JAK3; NF1; RUNX1; TET2 | 9 | 419.92 |
|  | FTE999 | *15* | 13.7 | FLT3; MLP; TET2 | 5 | 273.90 |
|  | FTE514 | *8* | 14.7 | CUX11; JAK2; NF1; SMC1A; TET2; TP53 | 8 | 1081.92 |
| *TP53* | FTE178 | *39* | 6.2 | CUX1 | 19 | 96.22 |
|  | FTE575 | *86* | 27.3 | U2AF1 | 3 | 1272.04 |
|  | FTE514 | *8* | 14.7 | CUX1; JAK2; NF1; SMC1A; SRSF2; TET2 | 8 | 1081.92 |
| *CUX1* | FTE178 | *39* | 6.2 | TP53 | 19 | 96.22 |
|  | FTE514 | *8* | 14.7 | TP53; JAK2; NF1; SMC1A; SRSF2; TET2 | 8 | 1081.92 |
| *DNMT3A* | FTE644 | *12* | 8.2 | ASXL1; FLT3; PHF6; RUNX1 | 9 | 704.75 |
|  | FTE193 | *75* | 8.9 | FLT3; IDH1; NRAS; SF3A1 | 3 | 739.50 |
| *KRAS* | FTE294 | *15* | 41.3 | GATA2 | 17 | 626.17 |
|  | FTE045 | *5* | 11.5 | ASXL1; TET2 | 4 | 775.25 |
| *NF1* | FTE118 | *56* | 2 | ASXL1; JAK3; RUNX1; SRSF2; TET2 | 9 | 419.92 |
|  | FTE514 | *8* | 14.7 | CUX11; JAK2; SMC1A; SRSF2; TET2; TP53 | 8 | 1081.92 |
| *RUNX1* | FTE118 | *56* | 2 | ASXL1; JAK3; NF1; SRSF2; TET2 | 9 | 419.92 |
|  | FTE644 | *12* | 8.2 | ASXL1; DNMT3A; FLT3; PHF6 | 9 | 704.75 |
| *WT1* | FTE855 | *76* | 27.6 | DNMT2A; IDH2; NPM1 | 4 | 1086.06 |
|  | FTE888 | *93* | 12.1 | FLT3; TET2 | 5 | 297.38 |

Mutations are listed from most to least common. Only mutations shared by 2 or more samples are shown. The 8:1 peak apoptosis represents the highest apoptotic leukemic cell count detected from the 8:1 E:T ratio in the live cell imaging apoptosis assays and the peak time is the hour when the 8:1 peak apoptosis was reached.
